# The unfinished agenda and inequality gaps in antenatal care coverage in Ethiopia

**DOI:** 10.1186/s12884-021-04326-y

**Published:** 2022-01-29

**Authors:** Sitota Tsegaye, Kalkidan Yibeltal, Haset Zelealem, Walelegn Worku, Meaza Demissie, Alemayehu Worku, Yemane Berhane

**Affiliations:** 1 Department of Nutrition and Behavioral Science, Addis Continental Institute of Public Health, Addis Ababa, Ethiopia; 2 Department of Reproductive Health and Population, Addis Continental Institute of Public Health, Addis Ababa, Ethiopia; 3 Department of Epidemiology, Georgia University School of Public Health, Athens, USA; 4 Department of Environmental Health, Addis Continental Institute of Public Health, Addis Ababa, Ethiopia; 5 Department of Global Health, Addis Continental Institute of Public Health, Addis Ababa, Ethiopia; 6 School of Public Health, College of Health Sciences, Addis Ababa University, Addis Ababa, Ethiopia; 7 Department of Epidemiology and Biostatistics, Addis Continental Institute of Public Health, Addis Ababa, Ethiopia

**Keywords:** Antenatal care, Household wealth, Maternal education, Residence, Inequality, Ethiopia

## Abstract

**Background:**

Antenatal care is an essential platform to provide all the necessary health interventions during pregnancy that aim to reduce maternal and newborn morbidity and mortality. Although the antenatal care coverage has been increasing in Ethiopia in the last two decades, the country has not been able to meet its own coverage target to date. Most pregnant women who initiated antenatal care also do not complete the full recommended follow up contacts. This study investigated the trend in coverage and the inequalities related to the use of antenatal care in Ethiopia.

**Methods:**

This study utilized data from five rounds of Demographic and Health Surveys (DHSs) conducted in Ethiopia in the period between 2000 and 2019. The DHS respondents were women in the age group 15-49 who had a live birth within the five years preceding the surveys. The outcome of interest for this study was antenatal care utilization coverage. We used concentration curve and concentration index to identify the inequalities using the World Health Organization recommended Health Equity Analysis Toolkit software. We did a regression analysis to identify the drivers of urban-rural inequalities.

**Result:**

The coverage trend for both initiating Antenatal care and completing the recommended four antenatal contacts showed a steady increase during 2000-2019. However, the coverages have not yet reached the national target and unlikely to meet targets by 2025. Although the economically better-off, urban and educated mother still have a better coverage, the inequality gaps within the wealth, residence and education categories generally showed significant reduction. Women in the lowest wealth quantile, those who were uneducated and those living in rural areas remained disadvantaged. Household economic status and maternal education was the stronger drivers of urban-rural inequalities.

**Conclusion:**

The Antenatal care coverage is lagging below the country’s target. Despite narrowing inequality gaps women from poor households, who are uneducated and residing in rural areas are still less likely to fully attend the recommended number of antenatal care contacts. Addressing these inequalities through a multisectoral efforts is critical to increase the chances of achieving the national antenatal care coverage targets in Ethiopia.

## Background

Antenatal care is a widely used strategy to deliver recommended health interventions during pregnancy in order to reduce maternal and newborn morbidity and mortality and improve newborn survival [1–3]. Ethiopia adopted focused antenatal care (FANC) model which was recommended by the World Health Organization’s (WHO) since 2002 [4, 5]. The focused antenatal care model recommends women to make at least four antenatal care (ANC) visits during their pregnancy [4, 6, 7].

The health care that a mother receives during pregnancy is important for the survival and well-being of both the mother and her baby [8]. Globally, more than 800 women are estimated to die daily from complications related to pregnancy and childbirth, countries in Sub-Saharan Africa account for the majority of deaths [9, 10]. The high maternal mortality ratios strongly correlate with low antenatal coverage [11]. ANC also provides opportunities to provide the necessary care to reduce low birth weight and improve newborns’ survival [3, 12].

In Ethiopia, the ANC coverage has been steadily increasing in the last two decades [10, 13]. However, both the initiation of ANC and completion of the recommended four contacts have been significantly lower than the target set for the nation [14]. The ANC utilization has been also significantly lower in some population group than others raising serious concerns of inequalities [15]. Equitable access to quality maternal health care is essential to improve maternal health outcomes and are widely advocated for [16]. In low-income countries, inequality by residential location (urban/rural), household wealth [17, 18], and maternal educational status [18, 19] are recognized as major impediments to achieving high coverage.

The failure to achieve national targets appears to have been deceived by the remarkable coverage improvements from one round of DHS to the next [10, 20]. Examining the trend with an eye on the target and identifying inequality gaps are necessary to inform decision making and to push harder to reach targets if and when necessary.

This study examined the trends in ANC/ANC 4+ national coverage and the inequality gaps in Ethiopia using a large representative sample from the Demographic and Health Surveys (DHS). The findings of this study provide empirical evidence for policy decisions to ensure equal access and utilization of ANC services in Ethiopia.

## Methods

### Study setting

Ethiopia has the second largest population in Africa with an estimated population of 112 million in 2019 [21]. Ethiopia, during the study period, has nine regional states and two city administrations. The country has a four-tier health care system with a broad base primary health care unit (PHCU) at the base. A typical PHCU consists of a health center and five-seven satellite health posts and is supposed to serve approximately 25,000 population. ANC services for all pregnant mothers is provided free of charge in all public health facilities. Several strategies including health promotion through the health extension workers were used to mobilize mothers to utilize maternal health services in Ethiopia.

### Study design and population

This paper was based on multiple EDHS surveys (cross sectional surveys) conducted in the period from 2000 to 2019. The surveys were conducted approximately in 5 years interval. The study population constitutes women in the age group 15-49 years. The main inclusion criterion was to be women who had at least one live birth in the 5 years preceding the demographic and health survey at each point.

### Sampling method

The sampling frame for the EDHS was prepared for each round based on the most recent population and housing census [13]. Such sampling strategy for DHS is standardized and applied in many low-and middle- income countries [22]. The sampling frame consists of a complete list of all enumeration areas (EA). An EA is a geographic area consisting of a convenient number of dwelling units which served as counting unit for the census. The frame file contains information about the location, the type of residence, and the number of residential households for each of the EAs [8, 13, 23–25]. A stratified two-stage cluster sampling procedure was used in order to select a nationally representative sample proportionate to the population size of each state in the country [13].

### Data collection

The DHSs collected data using a structured questionnaire consisting of several modules including household and women modules. Interviews were conducted face-to-face with eligible women at home by trained data collectors.

The data for this analysis were accessed by requesting the DHS program website (http://dhsprogram.com/data/available-datasets.cfm) after explaining the purpose of this study.

### Data analysis

The national coverages of initiation of antenatal care (ANC) and completion of the four recommended antenatal contacts (ANC 4+) were calculated by dividing the number of women who reported making ANC or ANC 4+ contacts by the total number of pregnant women in each survey, respectively. The projected coverage for 2025 was derived by calculating a smoothed yearly coverage. The annual increment between surveys was calculated by subtracting the coverage of the last survey from the current survey and then dividing by the number of years between the two surveys. The calculation for 2025 projection is shown below as an example:

$$\mathrm{Projection}\;\mathrm{for}\;2025\;=\;2019\;\mathrm{ANC}\;\mathrm{coverage}\;+\;\mathrm{Mean}\;\mathrm{smoothed}\;\mathrm{coverage}\;\mathrm{for}\;2000\;\mathrm{to}\;2019\;\mathrm{multiplied}\;\mathrm{by}\;\mathrm{the}\;\#\;\mathrm{of}\;\mathrm{years}\;\mathrm{between}\;2019\;\mathrm{and}\;2025$$

The projected coverage was compared to the target set for the year 2025 by the Ministry of Health (HSTP II). The equality analysis was done for household wealth quintiles (lowest or poorest, second, middle, fourth, highest or richest), maternal education (No education, Primary school, and Secondary school and above), and place of residence (rural/urban).

The equality analysis was done using the WHO HEAT model [26]. The toolkit produces concentration curve and concentration index to depict inequality. The concentration curve graphs were done using cumulative proportion of the ANC coverage against the cumulative proportion of the population ranked by living standards. As the WHO Toolkit did not yet integrate the 2019 mini-DHS data, the graphs were reconstructed in excel sheet using the XY (scatter) chart-type with data points connected by smoothed lines. The concentration index was calculated using the following formula:

$$\mathrm{Concentration}\ \mathrm{Index}\ \left(\mathrm{Conc}-\mathrm{I}\right)=\left(\mathrm{p}1\mathrm{L}2-\mathrm{p}2\mathrm{L}1\right)+\left(\mathrm{p}2\mathrm{L}3-\mathrm{p}3\mathrm{L}2\right)+\dots +\left(\mathrm{p}\mathrm{T}-1\mathrm{L}\mathrm{T}-\mathrm{p}\mathrm{T}\mathrm{LT}-1\right)$$

Where p is the cumulative proportion of people, L the cumulative proportion of ANC coverage, and T the number of deprivation groups.

The concentration index indicates the extent to which a health indicator, in this case the ANC coverage, is concentrated among the disadvantaged or the advantaged group by the equality variable of interest; in our case household wealth, maternal education status and type of residence. The concentration index is bounded between − 1 and + 1 (or − 100 and + 100); the value of zero indicates no inequality. Positive values indicate a concentration of the health indicator among the advantaged, while negative values indicate a concentration of the health indicator among the disadvantaged. When the concentration curve moves away from the line of equality it shows greater inequality in distribution of the outcome of interest (ANC utilization in this study).

To identify the drivers of rural-urban inequalities we performed a multivariate decomposition regression analysis for nonlinear response model. The regression analysis was done using Stata software version 14 from the 2016 EDHS data.

## Result

### Antenatal coverage trend (2000-2019)

**Fig. 1:**
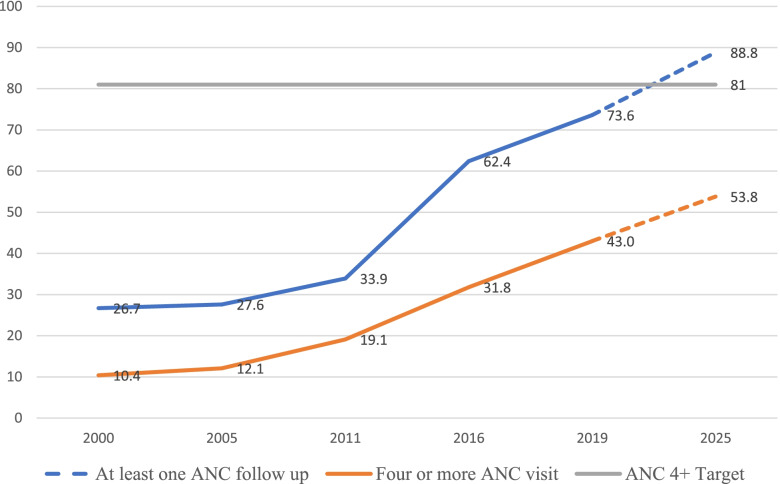
National Trend for at least one and 4 or more Antenatal Care visits Coverage trend 2000-2019 and projections for 2025 based on smoothed average for Ethiopia

The ANC4+ coverage trend steadily increased from 10.4% in 2000 to 43.0% in 2019. The projection for 2025 was 53.9%. The ANC initiation (at least one ANC contact) coverage trend curve also showed a steady increase from 26.7% in 2000 to 73.6% in 2019. The projection for 2025 was 88.8% (Fig. 1).

### Inequality gap analysis

**Fig. 2:**
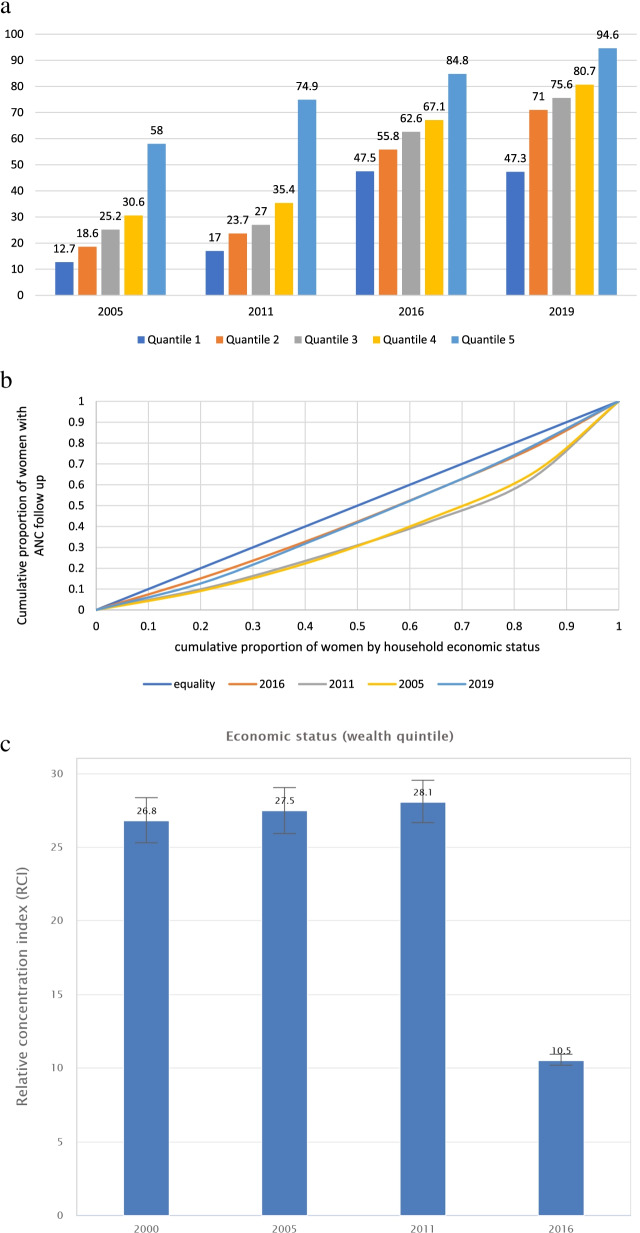
Antenatal care coverage inequality among wealth groups in Ethiopia, 2000-2019 **a** Coverage gaps between wealth strata; **b** Concentration curve and **c** Relative concentration index by household wealth quintile (2000-2016)

The inequality gap analysis by household wealth quantiles revealed that all the quantiles have made a steady and significant gains in coverage since 2000 (Fig. 2a). The concentration curve (Fig. 2b) and the relative concentration index (Fig. 2c) also showed a significant reduction in inequality gaps by wealth mainly because of the substantial increment in coverage in the lower wealth quintile groups.

**Fig. 3:**
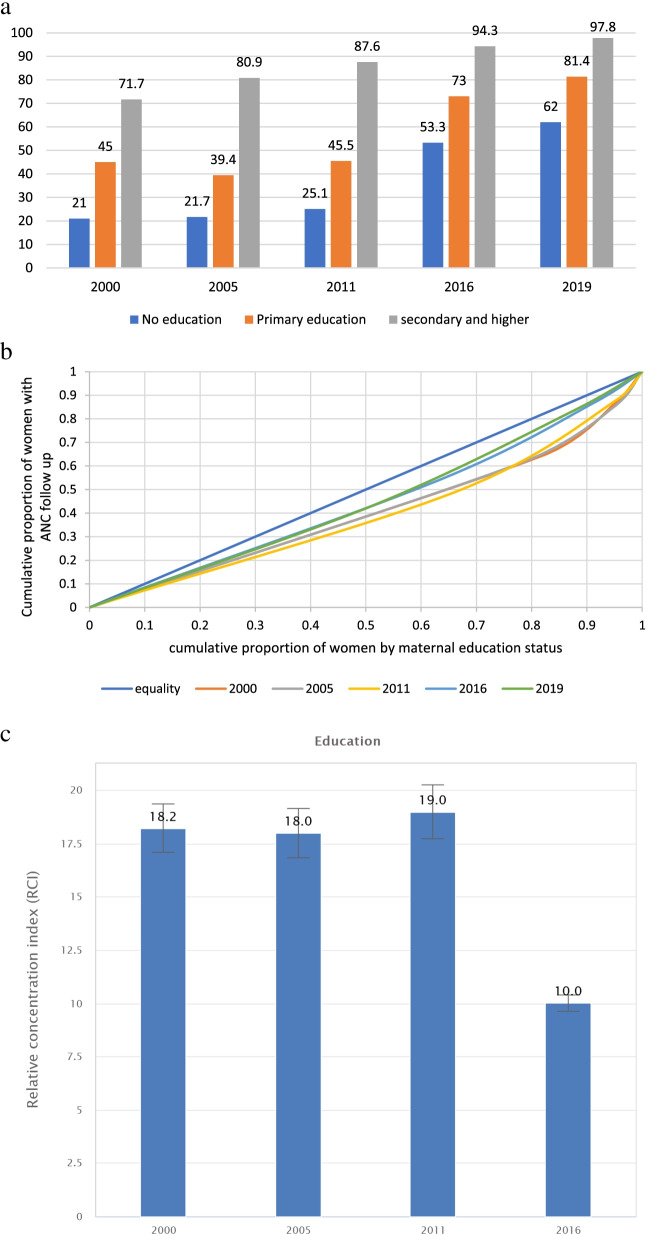
Antenatal care coverage inequality among education groups in Ethiopia, 4,242,000-2019 **a** Coverage gaps; **b** Concentration curve and **c** Relative concentration index by maternal education status (2000-2016)

A similar pattern was observed for the inequality analysis by educational status, which is steady and progressive gains in coverage (Fig. 3a). The concentration curve (Fig. 3b) and the relative concentration index (Fig. 3c) also showed a significant reduction in inequality gaps by maternal educational status.

**Fig. 4:**
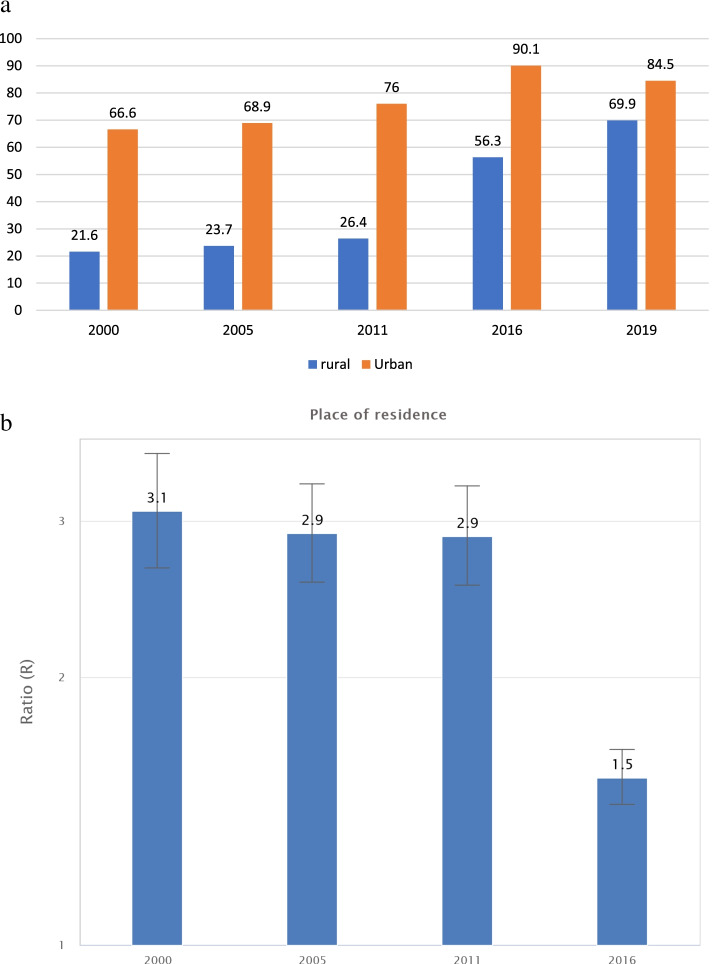
Antenatal care coverage inequality by residence area (rural Vs urban) in Ethiopia, 2000-2019 **a** Coverage gaps; **b** Ratio by place of residence (2000-2016)

The inequality gap by place of residence showed significant improvement in rural areas compared to urban areas (Fig. 4a). The inequality ratio between the urban and rural was also closing progressively mainly due to progress made in rural areas (Fig. 4b).

Even though the inequality gaps are narrowing between the advantaged and disadvantaged groups, the concentration curves in all parameters are below the diagonal line of equality (Figs. 2b and 3b).

According to the decomposition analysis the household economic status is the dominant contributing factor, contributing for 64%, for four or more ANC service utilization inequality by residency area. The maternal educational status was the second dominant driver, accounting for 28.48% of the contributing factor (Table 1).

**Table 1:** Logit decomposition of the four or more ANC visits service utilization inequality by area of resident. (Ethiopia, 2016)

**Due to difference in characteristics.**						
**ANC**	**Coef.**	**Std. Err.**	**z**	**P > z**	**95% Conf. Interval]**	**Pct.**
Age	.002767	.0010237	2.70	0.007	.00076063, .0047734	−.71782
Economic status	−.24693	.03045	− 8.11	0.000	−.30661, −.18725	64.058
Highest educational level	−.10979	.011865	− 9.25	0.000	−.13305, −.086536	28.482
Occupation	−.0002908	.0014452	− 0.20	0.841	−.0031234, .0025418	.07544
Mass media	−.0001329	.0030584	− 0.04	0.965	−.0061273, .0058615	.034477
Marital status	.00011897	.0017303	0.07	0.945	−.0032723, .0035103	−.030863
**Due to difference in coefficient**						
**ANC**	**Coef.**	**Std. Err.**	**z**	**P > z**	**95% Conf. Interval]**	**Pct.**
Age	−.2216	.12682	−1.75	0.081	−.47016, .026969	57.48
Economic status	−.064963	.060829	− 1.07	0.286	−.18419, .054261	16.853
Highest educational level	−.0072728	.0074648	− 0.97	0.330	−.021904, .0073582	1.8867
Occupation	.0023869	.003225	0.74	0.459	−.003934, .0087079	−.61922
Mass media	−.0023342	.0049994	− 0.47	0.641	−.012133, .0074647	.60553
Marital status	.01427	.021143	0.67	0.500	−.02717, .055711	− 3.702

## Discussion

The findings indicated that the national ANC and ANC4+ trend has been steadily increasing since 2000; however, the projected antenatal coverages are unlikely to meet the national targets for 2025, more notably for ANC 4+. The national target set for 2025 by the HSTP II is 81% [27]. The country has achieved significant reductions in the inequality gaps by household wealth, maternal education and place of residency. However, there still remain obvious gaps between the advantaged and disadvantaged groups in all three parameters observed in this study. Lower household economic status and maternal education are key drivers for urban-rural inequalities.

The increasing trend in initiation of ANC and completing the recommended four ANC can be explained by the expansion of new health facilities, the active engagement of community health workers (known as health extension workers in Ethiopia), and increased investment on maternal health services including fee exemption for such services in public health facilities in Ethiopia [28]. However, the target set for the past and current periods were not met which could be either due to ambitious targets setting or implementation challenges including supply side factors such as lack of medicines, unskilled health care professionals, poor attitude and unprofessional conduct of health workers [28, 29], and previous unpleasant experience at the health facilities hamper demand for services [11].

There are also many demand side factors that hinder initiation and completion of antenatal care. Its utilization is heavily hindered by far distance between residence and health facilities, lower educational status of both mothers and husbands, and poor economic status, [20]. Lack of transportation make repeated contacts to the health facilities very difficult for mothers, thus, demand is likely to decrease especially if the pregnancy is considered to be smooth by the mothers’ own assessment [14]. As the pregnancy advances mothers living far from health facilities may find it very difficult to walk long distances and fail to finish all the recommended ANC visits [30]. Financial and logistical problems are also substantial impediments to utilization of the ANC services in low-income settings [11]. ANC demand and utilization are also affected by factors such as age of the mother, previous history of ANC visit, parity, number of under-five children, and experiences during previous pregnancies and child birth [2, 14, 19, 31].

The significant reduction in the inequality gaps by household wealth was mainly as a result of higher percentage gain in the lower quintiles, which can be attributed the government’s pro-poor health policy and efforts to strengthen the grassroots level primary health care services [32]. However, the coverage in the lowest wealth quantile is still unacceptably low as also indicated by previous reports [32, 34]. This indicates the indirect costs to attend ANC are still prohibitive among the poor even the actual care is provided free of charge [35, 36]. Other important contributors to the disparity included lack of time to travel to a health facility; many poor mother may not have support at home for child care and other household chores [33, 37].

The inequality gap by place of residence is also closing for ANC coverage mainly due to a bigger improvement in rural areas compared to urban areas. Previously, health care facilities were more clustered in urban areas, in the last two decades health facilities have been built closer to rural residence [38], which is also related to increasing urbanization and establishment of small towns that can also provide accommodation for health workers [39]. Building health facilities closer to the rural population offers a better chance to utilize services even for mothers from the lower socioeconomic status [33, 40]. Urbanization also promote services utilization, women in urban areas use antenatal care service more frequently than those reside in rural areas due to ease of access [41]. In Ethiopia, the health infrastructure, distributing professional health care providers and human resource development at the primary health delivery points have shown significant improvement in rural areas in the last two decades, which was critical to closing the inequality by place of residence [42].

The inequality gaps by maternal education also revealed a significant reduction. Although coverage is still low in the lower educational groups it has shown remarkable improvement in the last two decades. This shows supporting women with lowest education to attend ANC can substantially reduce the gaps [17]. Educated women have a better socioeconomic status that offer them financial autonomy to seek medical care, in addition to a better access to information that allow them to make informed and positive decisions to seek care [14, 20].

The findings of this study have huge implication for practice and policy. Although efforts so far have bear fruits in terms increasing coverage, they were not matching the country’s ambition to make service accessible to the large majority of the population. Thus, it is important to consider policy options to reach more vulnerable and remotely located women. A one-size fit all approach need to be reconsidered to address the diversity of the population in terms of lifestyle (agrarian versus pastoralist) and residence (rural versus urban). The strength of this paper is usinWg the DHS data which has a large representative sample size collected on several points over a period of two decades. However, the EDHS data relied only on the ability of respondents to correctly recall and report past events without any means of further verification by the interviewers, we considered this recall bias as one of limitations of the study... Another strength of this study is the consideration given to past performance levels to make projections in to the future plan period.

## Conclusion

Despite a steady progress in antenatal coverage over the last 20 years the national coverage remained below the national target at all survey points. Even if there is significant improvement in the overall utilization of antenatal care in the country; the poor, uneducated and rural women remained constantly disadvantaged. We recommend expanding access to maternal health services at grassroot levels to further reduce the inequality gaps and increase coverage. Specific interventions that help overcome economic and educational disadvantages are critical. Such interventions may also require strong multisectoral efforts.

## Data Availability

Data for this study is available at the following website: http://dhsprogram.com/data/available-datasets.cfm

## Abbreviation

ANC: Antenatal Care
ANC 4+: Antenatal Care visits four or more.
ConI: Concentration Index
DHS: Demographic and Health Survey
EA: Enumeration Areas
EDHS: Ethiopia Demographic and Health Survey
FANC: Focused Ante Natal Care
HEAT: Health Equity Assessment Toolkit
HSTP: Health Sector Transformation Plan
MCH: Maternal and Child Health
MMR: Maternal Mortality Ratio
MOH: Ministry of Health
PHCU: Primary Health Care Unit
RCI: Relative Concentration Index
WHO: World Health Organization
